# Development and validation of a novel model based on hematological biomarkers for predicting risk factors and outcomes in cancer patients with carbapenem-resistant organism infection

**DOI:** 10.3389/fpubh.2026.1734259

**Published:** 2026-04-08

**Authors:** Bin Li, Xiaoyan Wen, Min Jing, Yuanye Qu, Junhua Zhang, Qingxia Xu, Shulin Chen, Ning Xue

**Affiliations:** 1 Department of Orthopedics, The First Affiliated Hospital of Zhengzhou University, Zhengzhou, China; 2 Central Sterilization Supply Department, The Guanghua Stomatological College of Sun Yat-sen University, Hospital of Stomatology, Sun Yat-sen University, Guangzhou, China; 3 Department of Clinical Laboratory, The Affiliated Cancer Hospital of Zhengzhou University & Henan Cancer Hospital, Zhengzhou, China; 4 State Key Laboratory of Oncology in South China, Guangdong Provincial Clinical Research Center for Cancer, Collaborative Innovation Center for Cancer Medicine, Sun Yat-sen University Cancer Center, Guangzhou, China

**Keywords:** cancer patients, carbapenem-resistant organism infection, LASSO regression, mortality, prognosis

## Abstract

**Background:**

This study aimed to develop and validate a novel model for predicting mortality risk in cancer patients with carbapenem-resistant organism (CRO) infections.

**Methods:**

Cancer patients with CRO infections in Henan Cancer Hospital between January 2022 and March 2024 were included in this retrospective study. LASSO regression was used to construct a novel model for predicting mortality in cancer patients with CRO infections. Receiver operating characteristic (ROC), decision curve analysis (DCA), and clinical impact curves (CIC) were used to assess the predictive ability and clinical utility of the prediction model.

**Results:**

A total of 417 cancer patients with CRO infections were included in the study. Fourteen factors were selected, including sample source, radiotherapy, blood culture, exposure to antibiotics after susceptibility testing, lymphocyte-to-monocyte ratio (LMR), lymphocyte-to-monocyte ratio (LMR), total protein (TP), blood urea nitrogen (BUN), calcium (CA), C-reactive protein (CRP), triglyceride (TG), procalcitonin (PCT), prothrombin time (PT), and thrombin time (TT), which were found to be associated with 30-day mortality in cancer patients with CRO infections. The areas under the ROC curve for the prediction model were 0.815 (95% CI: 0.767–0.857) and 0.801 (95% CI: 0.716–0.871) for the primary cohort and validation cohort, respectively. The models demonstrated good predictive accuracy, with *p*-values 0.479 and 0.786 on the Hosmer–Lemeshow test. DCA and CIC analyses confirmed the clinical utility of the prediction model.

**Conclusion:**

Our model could be used as an effective individualized risk prediction tool for clinicians. It would provide personalized risk assessments for cancer patients with CRO infections.

## Introduction

Cancer is one of the most significant threats to human health and continues to pose a major global public health challenge. According to cancer statistics for 2022, nearly 20 million new cancer cases and approximately 9.7 million cancer-related deaths were reported (1). Cancer patients are often susceptible to bacterial infections due to immunosuppression caused by the side effects of immunotherapy, chemotherapy, or surgery (2, 3). Additionally, cancer itself can impair host defenses due to malnutrition and compromised immune function (4). Therefore, bacterial infections are significant contributors to mortality in cancer patients.

Carbapenem-resistant pathogens are frequently identified due to the widespread use of carbapenems as empirical antibiotics with broad-spectrum activity. Inappropriate therapy may result in poor clinical outcomes. Data from the China Antimicrobial Surveillance Network (CHINET) indicate, that Acinetobacter exhibited a high prevalence of carbapenem resistance, approximately 66% in 2022 (5). Given this large number of carbapenem resistance, cancer patients infected with carbapenem-resistant bacteria may have higher probability experience poor clinical outcomes. Therefore, investigating the risk factors and prognosis of carbapenem-resistant infections in cancer patients is crucial. In current study, few relevant studies have been conducted. Moreover, these studies either focus solely on risk factors in patients with specific types of cancer, such as hematologic malignancies (6, 7), or emphasize a single infection source or pathogen (8–10). Furthermore, these studies primarily focused on clinical characteristics while neglecting important blood biochemical indicators (11).

LASSO regression is an effective tool for screening candidate factors prior to developing a prediction model. It achieves sparsity by introducing the L1 regularization term and is suitable for high-dimensional data analysis and variable screening. Many studies have widely used LASSO regression to establish models and to predict mortality risk in infected patients (12–14). Therefore, we developed a prediction model to investigate the risk factors and prognosis of cancer patients with multiple infections caused by carbapenem-resistant bacteria, with the aim of creating a prognosis model that could be more widely used in clinical practice.

## Materials and methods

### Patients and definitions

This retrospective study examined cancer patients with carbapenem-resistant organism (CRO) infections between January 2022 and March 2024 at The Affiliated Cancer Hospital of Zhengzhou University. The inclusion criteria were as follows: (a) only the first episode of infection per patient was included, (b) essential patient information was complete, and (c) the diagnosis of CRO infection was confirmed by susceptibility testing. This study focused on the risk of mortality within 30 days. The exclusion criteria were as follows: (a) patients with missing data, (b) patients without the survival outcomes. The outcome measured was all-cause mortality within 30 days following susceptibility testing. Follow-up data were obtained from medical records and telephone inquiries.

CRO infection was classified as hospital acquired if the sample culture was collected more than 48 h after admission and the patient showed no signs or symptoms of infection at admission. CRO infection was considered community-acquired if the sample culture was collected within 48 h of admission. Carbapenem resistance was defined as resistance to at least one of the following drugs: imipenem, meropenem, and ertapenem. Treatment was considered effective if empirical antibiotic therapy or exposure to antibiotics after susceptibility testing resulted in cultured bacteria being susceptible to at least one antibacterial agent *in vitro*. Respiratory tract infection samples included sputum, throat swabs, and bronchoalveolar lavage fluid.

### Data collection

Based on electronic medical record system, clinical data were collected on the following variables: gender, age, body mass index (BMI), sample source, types of cancer, bacterial species, parenteral nutrition, diabetes, type of indwelling needle, chemotherapy, radiotherapy, indwelling catheter, antifungal prophylaxis, body temperature, blood culture, empirical antibiotic therapy, exposure to antibiotics after susceptibility testing, infection source, C-reactive protein (CRP, mg/L), white blood cell count (WBC, ×10^9^/L), red blood cell count (RBC, ×10^9^/L), neutrophil count (NEU, ×10^9^/L), lymphocyte count (LY, ×10^9^/L), monocyte count (MO, ×10^9^/L), hemoglobin (HGB, g/L), platelet (PLT, ×10^9^/L), neutrophil-to lymphocyte ratio (NLR), lymphocyte-to-monocyte ratio (LMR), platelet-to-lymphocyte ratio (PLR), lymphocyte-to-CRP ratio (LCR), total bile acid (TBA, μmol/L), total bilirubin (TBIL, μmol/L), direct bilirubin (DBIL, μmol/L), total protein (TP, g/L), albumin (ALB, g/L), alanine aminotransferase (ALT, U/L), aspartate aminotransferase (AST, U/L), alkaline phosphatase (ALP, U/L), glutamyl transpeptidase (GGT, U/L), lactic dehydrogenase (LDH, U/L), hydroxybutyrate dehydrogenase (HBDH, U/L), creatine kinase (CK, U/L), creatine kinase-MB (CK-MB, U/L), creatinine (CRE, μmol/L), blood urea nitrogen (BUN, mmol/L), uric acid (UA, μmol/L), procalcitonin (PCT, ng/mL), Glucose (GLU, mmol/L), triglyceride (TG, mmol/L), cholesterol (CHO, mmol/L), high density lipoprotein (HDL, mmol/L), low density lipoprotein (LDL, mmol/L), very low density lipoprotein (VLDL, mmol/L), prothrombin time (PT, s), activated partial thromboplastin time (APTT, s), thrombin time (TT, s), fibrinogen (FIB, g/L), D-dimer(D2, mg/L), CRP/PCT ratio (CPT), lymphocyte-to-procalcitonin ratio (LPR), calcium (CA, mmol/L), systemic inflammation response index (SIRI) = NEU × MO/LYM (15), prognostic impact index (PNI) = ALB (g/L) + 5 × LYM × 10^9^/L (16), systemic immune-inflammation index (SII) = NEU × PLT/LYM (17). Moreover, the results of drug susceptibility tests of pathogens to commonly used antibiotics were also collected.

### Statistical analysis

All data were analyzed using R software (version 3.6.2) and SPSS (version 25.0, IBM, Chicago). LASSO regression (using ‘glmnet’ R package) was employed to screen the final factors and develop a prediction model for cancer patients with CRO infections, aimed at predicting the risk of 30-day mortality. In the LASSO model, 10-fold cross-validation and L1 regularization were applied to minimize the risk of overfitting and optimize the regression coefficients. Variables were tested with Wilcoxon test or Chi-squared test. Receiver operating characteristic (ROC), calibration curves, decision curve analysis (DCA), and clinical impact curves (CIC) were used to assess the predictive power of the model. The area under the curve (AUC) was calculated using the R package ‘survival ROC’. The CRO infection patients were divided into high-risk and low-risk groups based on the cut-off point, which obtained using the R package ‘survminer’. The Kaplan–Meier method was employed to evaluate overall survival (OS) in the high-risk and the low-risk groups. A *p*-value less than 0.05 was considered statistically significant, and all tests performed were two-tailed.

## Results

### Characteristics of cancer patients with CR

**Figure 1:**
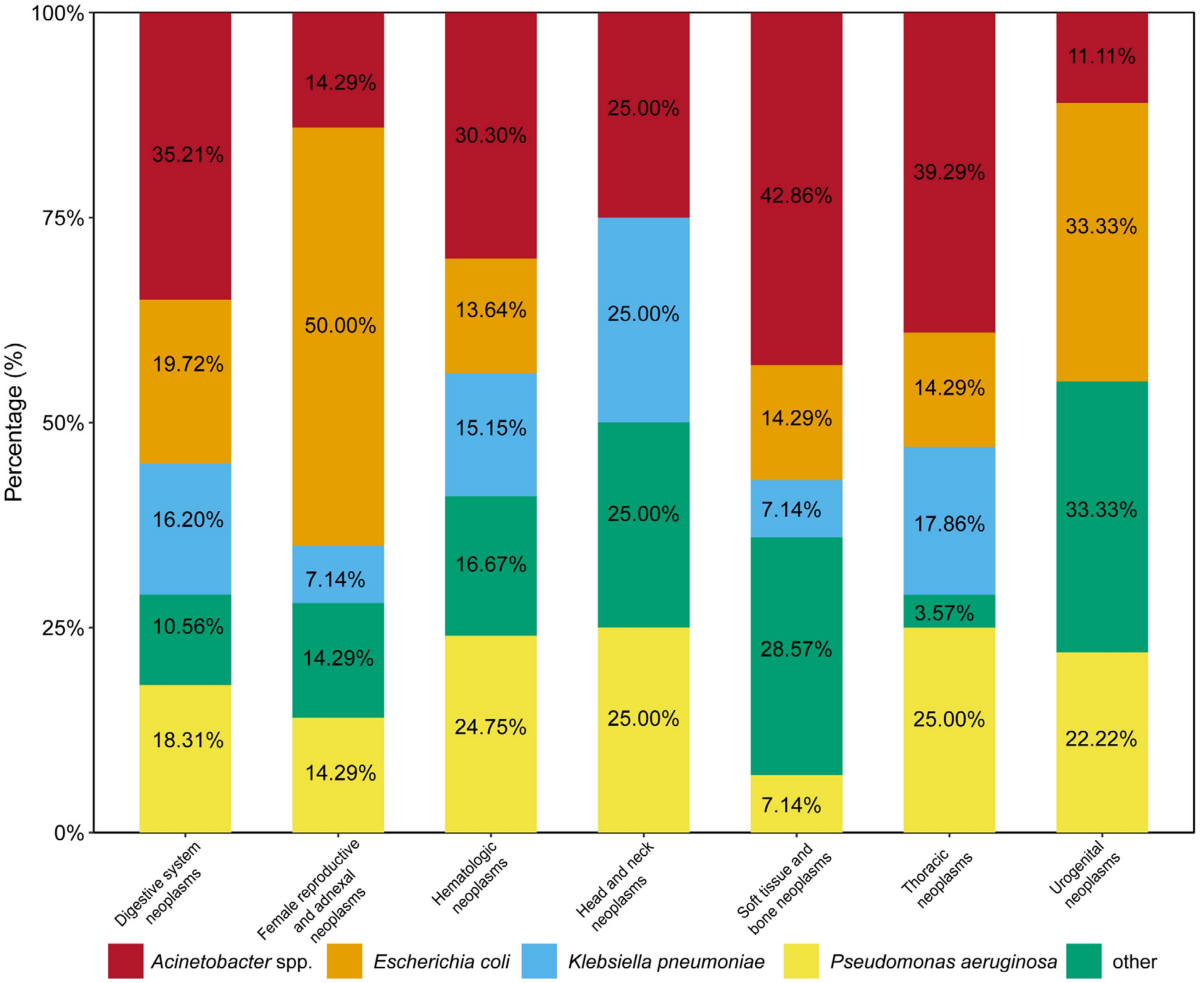
Percentages of carbapenem-resistant organism among different types of cancer patients.

A total of 417 cancer patients were enrolled in this retrospective study during the study period. There are 198 cases of hematologic neoplasms, 142 cases of digestive system neoplasms, 12 cases of head and neck neoplasms, 28 cases of thoracic neoplasms, 9 cases of urogenital neoplasms, 14 cases of soft tissue and bone neoplasms, and 14 cases of female reproductive and adnexal neoplasms. The percentage of each bacterial species in different type of cancers are shown in Figure 1. The patients were randomly divided into a primary cohort (*n* = 304) and validation cohort (*n* = 113). Table 1 presents the clinical characteristics and laboratory test results of the primary and validation cohorts. In the primary cohort, there were 177 females (58.22%) and 71 males (62.83%), with a mean age of 54.04 years (SD = 18.34). The most common source of infection samples was the respiratory tract (*n* = 140, 46.05%). In both cohorts, hematologic neoplasms were the most common cancer type (45.72% vs. 52.21%), followed by digestive system neoplasms (36.51% vs. 27.43%). Moreover, the 30-day mortality rates of cancer patients with CRO infection were 28.29 and 21.24% in the primary and validation cohort, respectively.

**Table 1:** Participant characteristics in the primary and validation cohort.

Characteristic	Primary cohort	Validation cohort	*p-*value
	*n* = (304)	*n* = (113)	
	No. (%) or mean ± SD	No. (%) or mean ± SD	
Gender			0.394^a^
Female	177 (58.22%)	71 (62.83%)	
Male	75 (41.78%)	42 (37.17%)	
Age (years)	54.04 ± 18.34	55.36 ± 17.19	0.656^b^
BMI (kg/m^2^)			0.471^a^
<18.00	48 (15.79%)	13 (11.50%)	
18.00–24.00	164 (53.95%)	61 (53.98%)	
>24.00	92 (30.26%)	39 (34.52%)	
Sample source			0.047^a^
Blood	23 (7.57%)	14 (12.39%)	
Respiratory tract	140 (46.05%)	61 (53.98%)	
Urine	28 (9.21%)	11 (9.73%)	
Excrement	29 (9.54%)	3 (2.65%)	
Drainage fluid	84 (27.63%)	24 (21.24%)	
Types of cancer			0.025^a^
Hematologic neoplasms	139 (45.72%)	59 (52.21%)	
Digestive system neoplasms	111 (36.51%)	31(27.43%)	
Head and neck neoplasms	10 (3.29%)	2 (1.77%)	
Thoracic neoplasms	15 (4.93%)	13 (11.50%)	
Urogenital neoplasms	8 (2.63%)	1 (0.88%)	
Soft tissue and bone neoplasms	8 (2.63%)	6 (5.31%)	
Female reproductive and adnexal neoplasms	13 (4.28%)	1 (0.88%)	
Bacterial species			0.581^a^
*Escherichia coli*	46 (15.13%)	24 (21.24%)	
*Klebsiella pneumoniae*	45 (14.80%)	18 (15.93%)	
*Pseudomonas aeruginosa*	68 (22.37%)	22 (19.47%)	
*Acinetobacter* spp.	101 (33.22%)	32 (28.32%)	
Other	44 (14.47%)	17 (15.04%)	
Parenteral nutrition			0.087^a^
Yes	210 (69.08%)	68 (66.67%)	
No	94 (30.92%)	45 (39.82%)	
Diabetes			0.654^a^
Yes	248 (81.58%)	90 (79.65%)	
No	56 (18.42%)	23 (20.35%)	
Chemotherapy			0.788^a^
Yes	98 (32.24%)	38 (33.63%)	
No	206 (67.76%)	75 (66.37%)	
Type of indwelling needle			0.440^a^
Central venous catheter	168 (55.26%)	69 (61.06%)	
PICC	104 (34.21%)	36 (31.86%)	
Short term indwelling needle	32 (10.53%)	8 (7.08%)	
Radiotherapy			0.395^a^
Yes	(90.46%)	99 (87.61%)	
No	29 (9.54%)	14 (12.39%)	
Indwelling catheter			0.331^a^
Yes	252 (82.89%)	89 (78.76%)	
No	52 (17.11%)	24 (21.24%)	
Antifungal prophylaxis			0.047^a^
Yes	173 (56.91%)	52 (846.02%)	
No	131 (43.09%)	61 (53.98%)	
Body temperature			0.691^a^
≤38.50	266 (90.79%)	104 (92.04%)	
>38.50	28 (9.21%)	9 (7.96%)	
Blood culture			0.459^a^
Positive	239 (78.62%)	85 (75.22%)	
Negative	65 (21.38%)	28 (24.78%)	
Empirical antibiotic therapy			0.167^a^
Effective treatment	243 (79.93%)	97 (85.84%)	
Ineffective treatment	61 (20.07%)	16 (14.16%)	
Exposure to antibiotics after susceptibility testing			0.268^a^
Effective treatment	123 (40.46%)	39 (34.51%)	
Ineffective treatment	181 (59.54%)	74 (65.49%)	
Infection source			0.218^a^
Hospital acquired	86 (28.29%)	39 (34.51%)	
Community acquired	218 (71.71%)	74 (65.49%)	
WBC (10^9^/L)	9.74 ± 21.76	8.55 ± 11.70	0.938^b^
RBC (10^9^/L)	2.97 ± 0.69	3.00 ± 0.62	0.917^b^
Neutrophils (10^9^/L)	7.01 ± 14.28	6.45 ± 7.16	0.910^b^
Lymphocyte (10^9^/L)	1.04 ± 3.55	1.56 ± 8.88	0.561^b^
Monocytes (10^9^/L)	0.88 ± 6.03	0.45 ± 1.34	0. 430^b^
HGB (g/L)	90.56 ± 19.55	91.67 ± 19.37	0.922^b^
Platelet (10^9^/L)	141.63 ± 121.548	137.34 ± 118.73	0.776^b^
NLR	15.50 ± 36.81	16.71 ± 33.49	0.985^b^
LMR	7.88 ± 38.10	5.21 ± 16.14	0.644^b^
PLR	491.22 ± 1008.65	568.33 ± 1107.79	0.633^b^
LCR	0.08 ± 0.25	0.11 ± 0.45	0.747^b^
TBA (μmol/L)	6.70 ± 12.17	7.06 ± 14.26	0.629^b^
TBIL (μmol/L)	27.79 ± 49.82	23.65 ± 33.04	0.748^b^
DBIL (μmol/L)	18.73 ± 38.59	15.34 ± 26.27	0.779^b^
TP (g/L)	58.35 ± 9.31	57.88 ± 8.65	0.739^b^
ALB (g/L)	34.29 ± 5.33	34.10 ± 5.44	0.699^b^
ALT (U/L)	37.16 ± 108.45	24.50 ± 26.92	0.948^b^
AST (U/L)	38.44 ± 127.17	28.08 ± 26.921	0.984^b^
ALP (U/L)	126.14 ± 132.41	120.42 ± 93.33	0.573^b^
GGT (U/L)	97.42 ± 157.54	95.05 ± 139.80	0.709^b^
LDH (U/L)	274.0.97 ± 242.99	270.56 ± 241.52	0.613^b^
HBDH (U/L)	222.80 ± 209.35	219.25 ± 216.87	0.550^b^
CK (U/L)	45.45 ± 59.41	50.58 ± 67.66	0.317^b^
CK-MB (U/L)	13.26 ± 9.69	13.89 ± 9.82	0.575^b^
CRP (mg/L)	76.39 ± 70.98	69.20 ± 62.50	0.550^b^
CRE (μmol/L)	68.23 ± 74.60	61.44 ± 32.29	0.285^b^
BUN (mmol/L)	6.88 ± 6.03	7.07 ± 5.46	0.260^b^
UA (μmol/L)	189.92 ± 117.09	185.04 ± 97.91	0.830^b^
PCT (ng/mL)	1.42 ± 5.34	1.60 ± 4.63	0.202^b^
GLU (mmol/L)	6.26 ± 2.39	6.38 ± 2.81	0.834^b^
TG (mmol/L)	1.75 ± 1.49	1.70 ± 1.24	0.666^b^
CHO (mmol/L)	4.03 ± 1.40	3.99 ± 1.19	0.906^b^
HDL (mmol/L)	1.03 ± 0.42	1.02 ± 0.40	0.878^b^
LDL (mmol/L)	2.54 ± 1.11	2.45 ± 0.90	0.802^b^
VLDL (mmol/L)	0.54 ± 0.57	0.53 ± 0.45	0.557^b^
PT (s)	14.19 ± 2.05	14.36 ± 2.12	0.315^b^
APTT(s)	34.74 ± 7.90	35.59 ± 7.55	0.364^b^
TT (s)	16.13 ± 1.71	16.09 ± 2.14	0.372^b^
FIB (g/L)	3.68 ± 1.32	3.66 ± 1.52	0.759^b^
D2 (mg/L)	2.12 ± 3.74	1.72 ± 2.08	0.453^b^
CPT	458.53 ± 936.87	362.11 ± 641.55	0.159^b^
LPR	10.64 ± 27.03	6.98 ± 17.33	0.255^b^
SII	1939.26 ± 3020.43	2574.67 ± 6046.68	0.908^b^
CA (mmol/L)	2.11 ± 0.20	2.17 ± 0.26	0.206^b^
SIRI	9.56 ± 38.34	6.06 ± 11.15	0.979^b^
PNI	39.49 ± 17.57	41.90 ± 45.53	0.585^b^

a Using Chi-squared test.

b Using Wilcoxon test.

PICC, peripherally inserted central catheter; BMI, body mass index; WBC, white blood cell; RBC, red blood cell; HGB, hemoglobin; NLR, neutrophil/lymphocyte ratio; LMR, lymphocyte/monocyte ratio; PLR, platelet/lymphocyte ratio; CRP, C-reactive protein; LCR, lymphocyte/CRP; TBA, total bile acid; TBIL, bilirubin total; DBIL, direct bilirubin; TP, total protein; ALB, albumin; ALT, alanine aminotransferase; AST, aspartate aminotransferase; ALP, alkaline phosphatase; GGT, glutamyl transpeptidase; LDH, lactic dehydrogenase; HBDH, hydroxybutyrate dehydrogenase; CK, creatine kinase; CKMB, creatine kinase-MB; CRE, creatinine; BUN, blood urea nitrogen; UA, uric acid; PCT, procalcitonin; GLU, Glucose; TG, triglyceride; CHO, cholesterol; HDL, high density lipoprotein; LDL, low density lipoprotein; VLDL, very low density lipoprotein; PT, prothrombin time; APTT, activated partial thromboplastin time; TT, thrombin time; FIB, fibrinogen; D2, D-Dimer; CPT, CRP/PCT; LPR, lymphocyte/PCT ratio; CA, calcium; SII, systemic immune-inflammation index; SIRI, systemic inflammation response index; PNI, prognostic impact index.

### Prediction model for the risk of 30-day mortality

**Figure 2:**
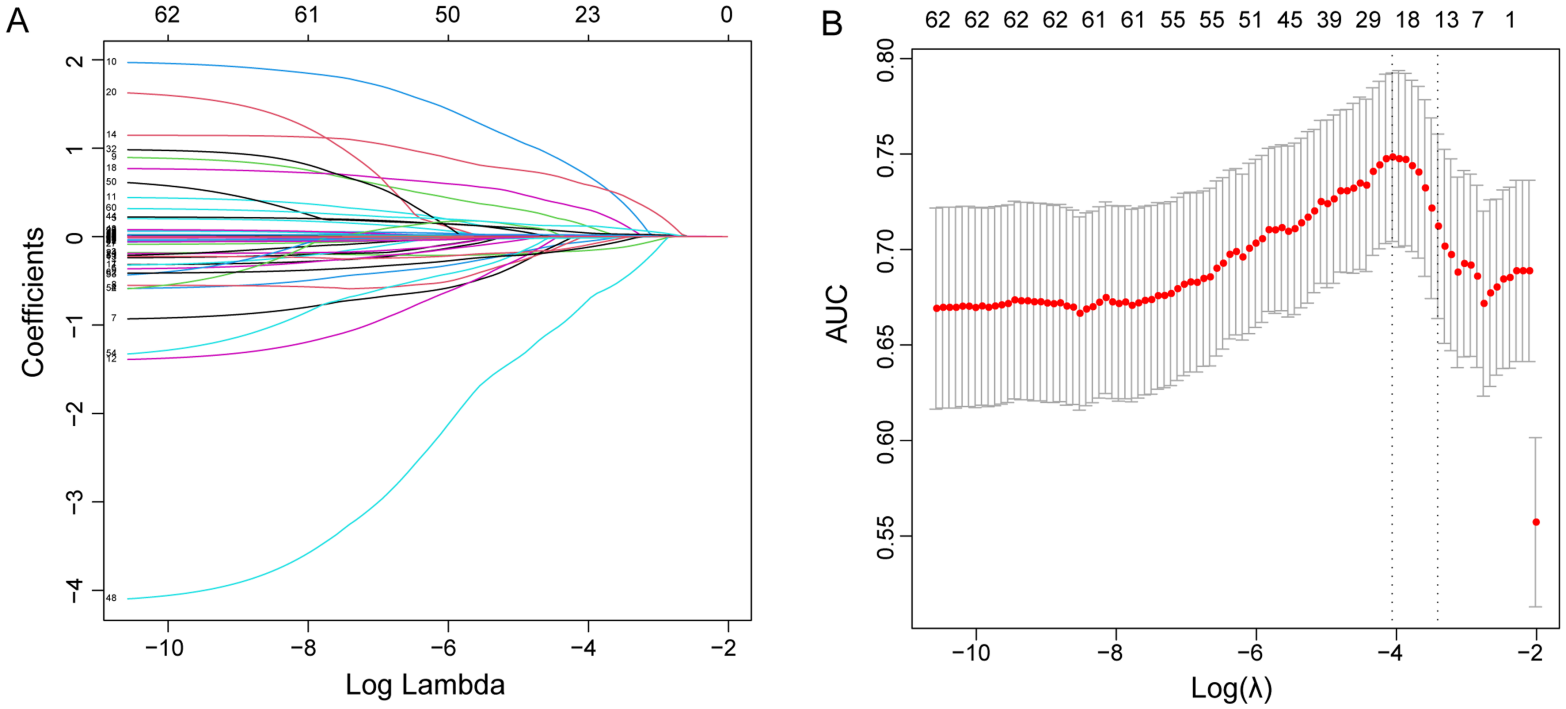
LASSO regression coefficient profiles of the 63 factors **(A)**. Ten-fold cross-validation via minimum criterion of LASSO regression **(B)**.

In the primary cohort, LASSO regression was used to select candidate factors from 63 clinical characteristics and laboratory tests. A total of 14 factors were selected, including sample source, radiotherapy, blood culture, exposure to antibiotics after susceptibility testing, LMR, LPR, TP, BUN, CA, CRP, TG, PCT, PT, and TT. Based on these factors, a cost-effective prediction model was constructed that demonstrated better performance. Figure 2A shows the trajectory intervals of each *λ*. The optimal lambda (*λ* = 0.03299747), indicated by the right dotted line, was Lambda.1se for these 14 factors with non-zero coefficients (Figure 2B). Finally, a prediction model was constructed based on these 14 factors. The computational formula was as follows: Risk score = − 1.294 × sample source + 0.308 × radiotherapy + 0.428 × blood culture + 0.097 × exposure to antibiotics after susceptibility testing + 0.004 × LMR – 0.001 × LPR – 0.008 × TP + 0.026 × BUN – 0.425 × CA + 0.006 × CRP – 0.002 × TG + 0.014 × PCT + 0.083 × PT – 0.029 × TT (We assigned the categorical variables as numeric variables. Sample source: 1, blood, 2, respiratory tract, 3, urine, 4, excrement, 5, drainage fluid. Radiotherapy: 1, yes, 0, no. Blood culture: 1, positive, 0, negative. Exposure to antibiotics after susceptibility testing: 1, effective treatment, 0 ineffective treatment).

### Prediction model evaluation

**Figure 3:**
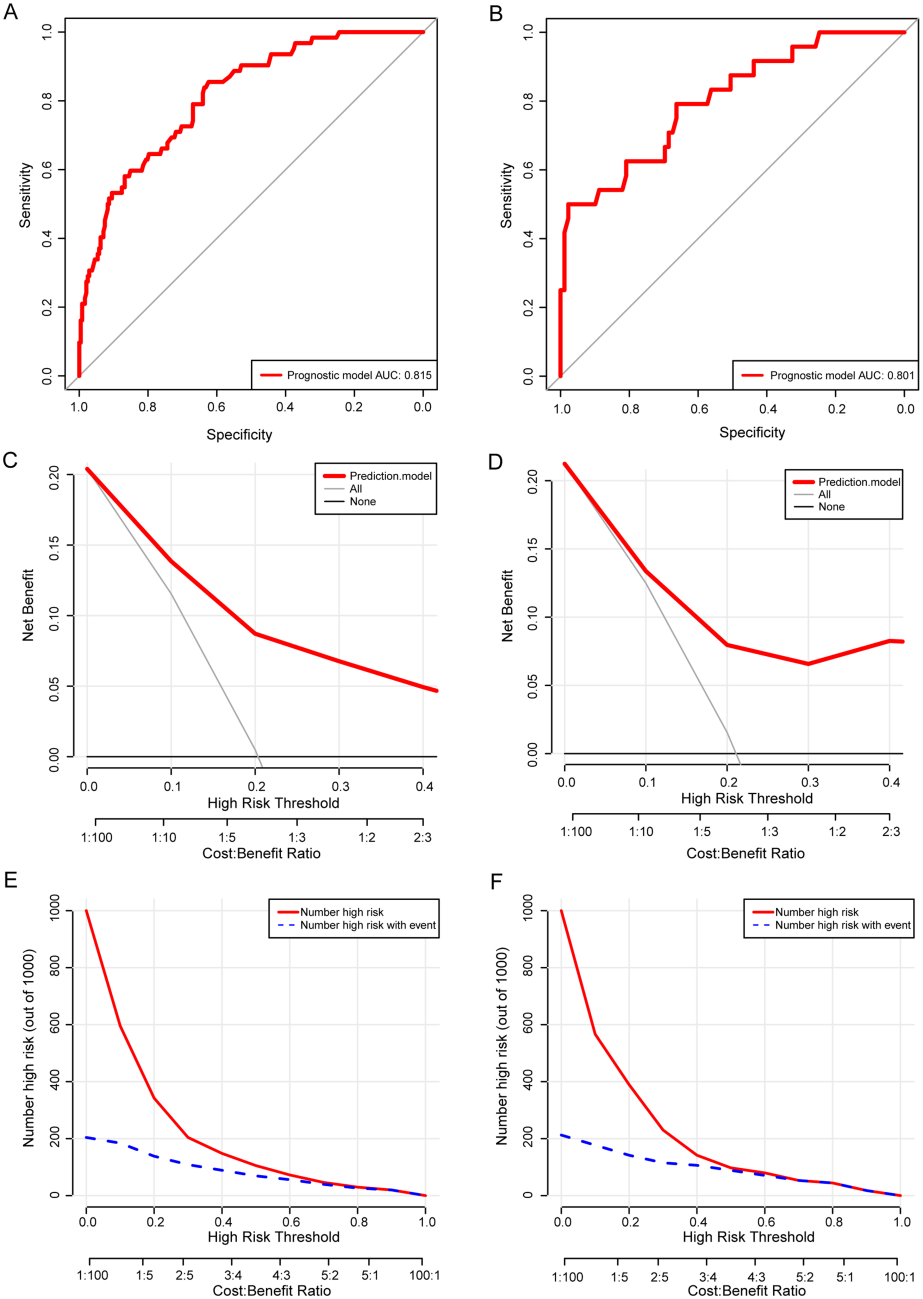
Receiver operating characteristic curve (ROC), decision curve analysis (DCA), and clinical impact curves (CIC) of the prediction model for predicting 30-day mortality of cancer patients with CRO infection in the primary cohort **(A,C,E)** and the validation cohort **(B,D,F)**.

In both the primary and validation cohort, ROC, DCA, and CIC were used to evaluate the predictive ability and clinical utility of the prediction model in CRO patients. The AUC of the prediction model was 0.815 (95% CI: 0.767–0.857) in the primary cohort and 0.801 (95% CI: 0.716–0.871) in the validation cohort (Figures 3A,B). DCA demonstrated that the nomogram offers superior net benefit for predicting 30-day mortality in cancer patients with CRO infections (Figures 3C,D). Clinical impact curves (CIC) demonstrated similar results in both the primary cohort and validation cohorts (Figures 3E,F). The red curves represent the number of cancer patients classified as high-risk by the prediction model at each threshold probability. The blue curves represent the number of true high-risk patients at each threshold probability.

### Stratification analysis of the prediction model

**Figure 4:**
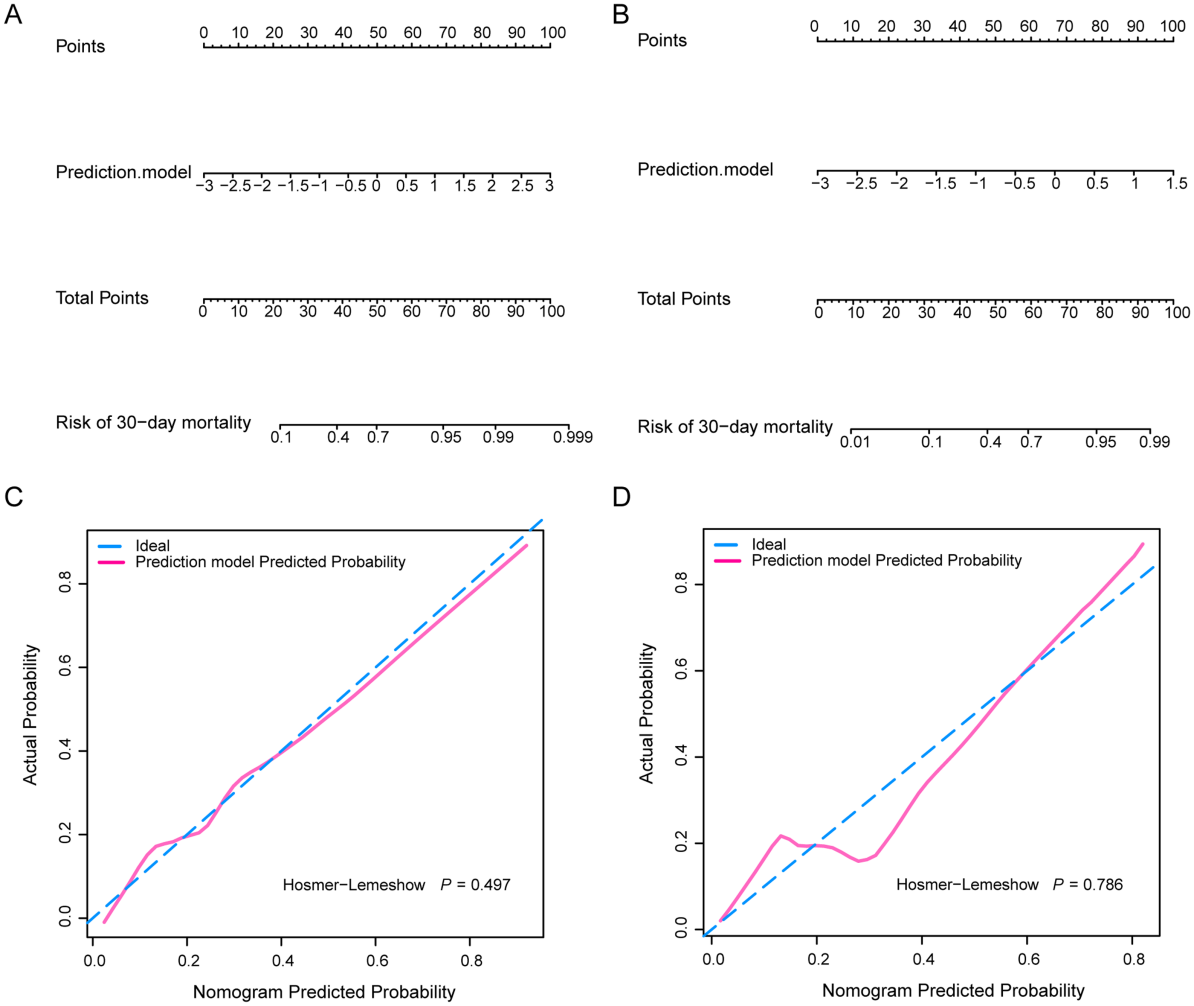
The nomograms **(A,B)** and the calibration curves **(C,D)** of the nomogram for predicting 30-day mortality of cancer patients with CRO infection in both two cohort **(C,D)**.

**Figure 5:**
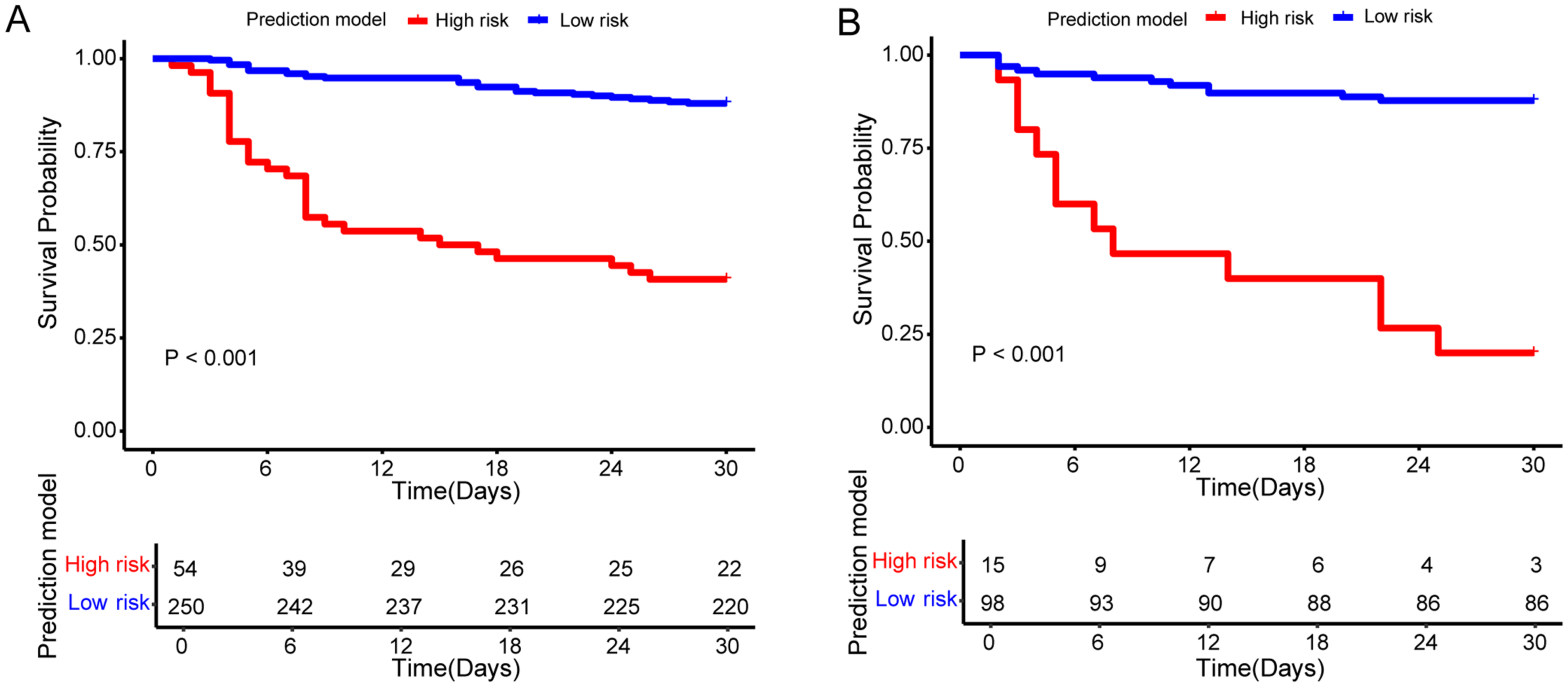
The levels of BUN, CA, CRP, LMR, LPR, PCT, PT, TG, TP, and TT for the high-risk and low-risk groups in the primary **(A)** and validation cohort **(B)**. * indicate *p* < 0.05, ** indicate *p* < 0.01, *** indicate *p* < 0.001, ns indicate no statistical significance.

**Figure 6:**
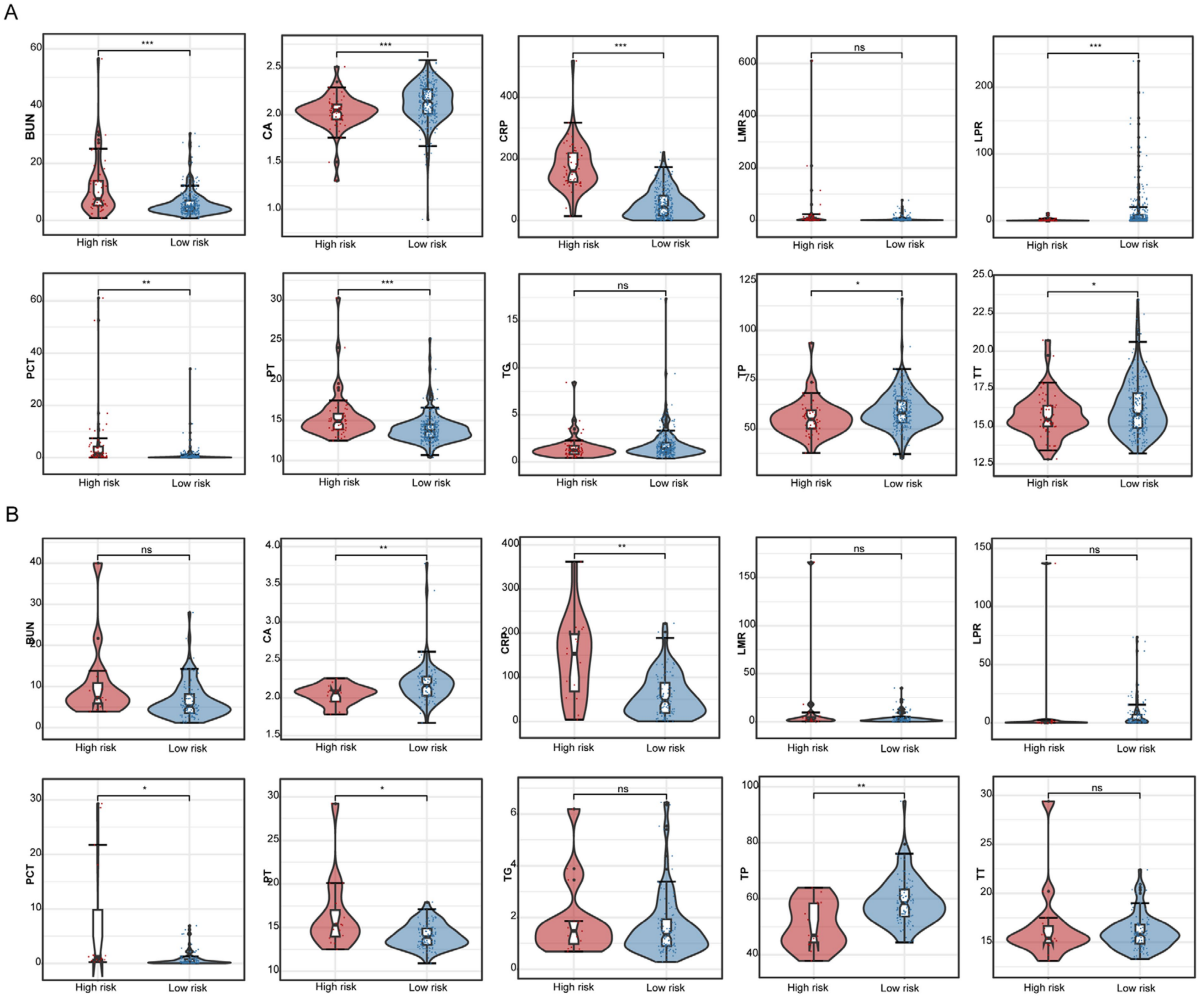
Kaplan–Meier curves of high-risk group and low-risk group for the prediction model in the primary **(A)** and validation cohort **(B)**.

Based on the prediction model, we developed a nomogram to easily estimate the mortality of cancer patients with CRO infections (Figures 4A,B). The calibration curves showed \high consistency between the actual 30-day mortality and the predicted 30-day mortality in both the primary and validation cohorts (Figures 4C,D). The *p*-value of the Hosmer–Lemeshow test were 0.497 and 0.786, indicating good discrimination. According to the optional cut-off value of the risk score (−0.82) in the prediction model, the CRO patients were divided into the high-risk group (≤ − 0.82) and the low-risk group (> − 0.82). The 30-day mortality rate in the high-risk group was significantly higher than that in the low-risk group in both cohorts (*p* < 0.001) (Figure 5). The levels of CA, CRP, PCT, PT, and TP were significant different between high-risk group and low-risk group in both the primary and validation cohort (*p* < 0.05). However, there was no significant difference in the levels of LMR and TG between high-risk and low-risk groups in both two cohorts (Figure 6).

## Discussion

Cancer patients are more susceptible to infections for various reasons, including surgery, malnutrition, immunosuppression, and the presence of indwelling devices (18, 19). In this study, the most numerous of cancer patients with CRO infections are patients with hematologic neoplasms and patients with digestive system neoplasms. The possible reason is that patients with hematologic neoplasms often have immunosuppression, while patients with digestive system neoplasms mostly receive surgical treatment. There is an urgent need to promptly and accurately identify high-risk cancer patients with CRO infections, which can help delay disease progression and optimize pharmacological management strategies. Moreover, accurate identification of the high-risk patients can further assist physicians in standardizing antibiotic use and enhancing awareness of infection prevention. This study systematically analyzed the clinical characteristics and laboratory test results of cancer patients with CRO infections. Furthermore, based on 63 clinical characteristics and laboratory tests, we developed a prediction model to predict the 30-day mortality rate for cancer patients with CRO infections.

In this study, LASSO regression analysis was used to identify potential factors from 63 variables. Ultimately, 14 factors were selected to construct the prediction model, including sample source, radiotherapy, blood culture, exposure to antibiotics after susceptibility testing, LMR, PLR, TP, BUN, CA, CRP, TG, PCT, PT, and TT. Summing the scores of each factor, which corresponds to the predicted probability of the clinical event, results in a cumulative total score that indicates the likelihood of a clinical event. The ROC analysis showed that the prediction model had good predictive accuracy. Moreover, the DCA and CIC results showed a greater net benefit for predicting 30-day mortality in cancer patients with CRO infections. According to the risk score of the prediction model, all cancer patients were divided into high-risk and low-risk groups. The high-risk group had a higher mortality rate than the low-risk group in the primary and validation cohorts (*p* < 0.001).

The present study not only elucidated the prognostic factors associated with several clinical characteristics but also included multiple blood biomarkers, which offer several advantages, such as ease of acquisition, low cost, and accuracy. The underlying mechanisms that explain the prognostic value of these biomarkers in infections are their close association with inflammation and the nutritional status of the patients. LMR has been found useful for identifying the susceptibility to malaria and distinguishing bacterial infections from viral infections in patients with fever (20, 21). Other studies have demonstrated that the inflammatory factors of LMR serve as useful prognostic markers for deep neck infections and complicated acute appendicitis in children (22). LMR has also been shown to have a significant predictive value in the mortality of COVID-19 patients (23). TP, BUN, and TG can directly reflect the nutritional status of patients. BUN reflects the status of protein breakdown in patients, as it is the primary end product of protein metabolism. Accordingly, elevated BUN levels are associated with mortality in critically ill patients and closely related to 30-day mortality in patients with sepsis (24, 25). Calcium homeostasis plays a crucial role in maintaining various physiological processes. It has been reported that serum calcium levels are associated with the risk of stroke-associated infections (26). Hypocalcemia is also commonly observed in septic patients caused by various mechanisms (27, 28). CRP is a nonspecific inflammatory biomarker (29). PCT has high sensitivity and specificity and helps determine the presence of bacterial infections (30). The combined detection of CRP and PCT has broad clinical applications in the diagnosis and prognosis evaluation of fracture-related infection (31). Cancer patients exhibited an altered coagulation state and have reduced immune function. Therefore, cancer patients are more susceptible to infections and coagulation dysfunction (such as APTT, PT, TT) can occur following infection (32).

This study has a few differences and advantages compared with other relevant studies. This study included multiple types of cancer instead of one specific type of cancer, which may make it have a wider range of uses. Besides, this study included various infection sources or pathogens instead of only emphasize a single infection source or pathogen. Such difference may enable the current study to better cope with complex clinical situations. Furthermore, our study integrated both clinical characteristics and multiple important blood biochemical indicators. These indicators have several advantages, such as routine detection, easy acquisition, and low cost so that they should not be neglected. This study has several drawbacks. First, as a single-center retrospective study, potential biases cannot be entirely excluded. Second, although the overall sample size is relatively large, the sample size for each individual tumor type was small. Therefore, our next objective is to conduct a multi-center study with a larger-sample size to optimize the prediction model.

## Conclusion

In this study, we developed a novel prediction model based on clinical characteristics and laboratory test data. For clinicians, this model may help them identify high-risk cancer patients and take appropriate measures to improve the therapeutic effect in cancer patients with CRO infections.

## Data Availability

The raw data supporting the conclusions of this article will be made available by the authors, without undue reservation.
